# A biomass derived porous carbon for broadband and lightweight microwave absorption

**DOI:** 10.1038/s41598-019-54104-2

**Published:** 2019-12-09

**Authors:** Zhu Zhang, Huanqin Zhao, Weihua Gu, Lieji Yang, Baoshan Zhang

**Affiliations:** 1 School of Electronic Science and Engineering, Nanjing University, Nanjing, 210093 P. R. China; 2 College of Materials Science and Technology, Nanjing University of Aeronautics and Astronautics, Nanjing, 211100 P. R. China

**Keywords:** Electronic devices, Porous materials, Synthesis and processing

## Abstract

With the continuous progress of science and technology, the traditional magnetic material is no longer able to meet the new complex electromagnetic (EM) environment due to its high bulk density. Therefore, the novel excellent EM absorber with the feature of thin thickness, low density, broad absorption bandwidth and strong absorption intensity is highly desired. Herein, we fabricated a porous carbon with ultrahigh porosity through a facile KOH activation from biomass waste pumpkin seed shell for lightweight EM wave absorption application. By optimizing the porous structures, the strong absorption intensity of −50.55 dB is achieved at thin thickness of 1.85 mm under low filler content of only 10 wt %. More interestingly, a broad frequency bandwidth of 7.4 GHz could cover the whole Ku band. These outstanding microwave absorption performances, couple with low cost ingredients and ease of fabrication process enable the porous carbon framework as the next generation promising candidate for lightweight and remarkable EM absorber.

## Introduction

With the continuous progress of science and technology, the problem of electromagnetic wave (EMW) pollution is increasingly prominent^1^. The electromagnetic wave not only pose a threat to national defense security, but also cause interference to civil facilities such as aviation systems and sophisticated electronic components. Therefore, it is very important to develop a high-performance absorbing material. Evaluating the performance of a wave-absorbing material generally takes into account such criteria as thickness, weight, loss capacity and bandwidth^2^. Usually, a lightweight and broadband microwave absorption material is very desirable. Thanks to the great virtues of controllable dielectric parameters, low density and chemical stability, multifarious carbon materials, such as graphene^3^, carbon nanotubes^4^, carbon black^5^, and porous carbon^6^ have been widely studied for EMW absorption. Recent researches mainly focus on the carbon/magnetic composites, unfortunately, the synthesis process of such material is complex and expensive, which cannot be put into practical application. Herein, we turn to the synthesis of a substantial and convenient single component carbon material.

Biomass, as a renewable resource, have some natural advantages in the preparation of special structural materials. Low doping, structure harmonic and single components are its inherent highlights. Recently, more and more researches have studied biomass-derived carbons using lotus seedpod^7^, silk^8^, shiitake^9^, willow leaves^10^, lignite^11^, pollens^12^, watermelon^13^, human hairs^14^, catkins^15^, macadamia shell^16^, pomelo peels^17^, etc. It’s wildly accepted that, adjusting the morphology and modifying the surface is important to improve the EMW loss ability. Herein, a lot of works had been done to explore different morphology of carbon materials toward EMW absorption. For example, Qiang *et al*. designed a mesoporous carbon sphere with a yoke-shell configuration via a “coating-coating-etching” approach^18^, achieving reflection loss (RL) of −39.4 dB at sample thickness 1.85 mm. Yin’s group fabricated mesoporous carbon spheres with hollow structures covering 8 GHz bandwidth at sample thickness of 2.15 mm^19^. Xu and his co-workers prepared mesoporous carbon hollow microspheres with red blood cell-like shapes through a modified Stober approach under a thermal decomposition and etching process. A minimum RL value of −59.7 dB and effective absorption bandwidth more than 3 GHz from 300 to 523 K was achieved^20^. Bi *et al*. synthesized the highly ordered porous carbon with 3D forms, which performed 4.5 GHz effective bandwidth under only 5% filler ratio^21^. These researches have made great progress, but commercial production cannot be guaranteed in terms of resource utilization and cost control, which greatly restricts the direct application in EMW absorption.

Here, we use shell of pumpkin seeds (SPS) with one million tons yield in China as the raw material^22^. Thus, there is no concern to worry about the short resources from primary product, which is exactly a prominent advantage for bio-carbon. Through KOH activation, a 3D connected network could be obtained. According to previous work, this kind of 3D network structure would lead to strong conduction loss^23^. Furthermore, by regulating graphitization of the bio-carbon, complex permittity of as-prepared samples could be easily adjusted. In addition, the morphology of this derived carbon in this work could also be controlled through the degree of activation. Combining these two strategy, a bio-carbon with a maximum effective absorption bandwidth of 7.4 GHz at a thickness of 2.6 mm was achieved. It should be pointed out that the filling ratio of the derived carbon is only 10 w%, in addition, the maximum reflection loss (RL) could reach to −50.55 dB. Therefore, this kind of porous carbon (PC) conforms to the characteristics of thin, lightweight, wide bandwidth and strong RL capacity for microwave absorption, which will have great application prospects in the future.

## Results

**Figure 1:**
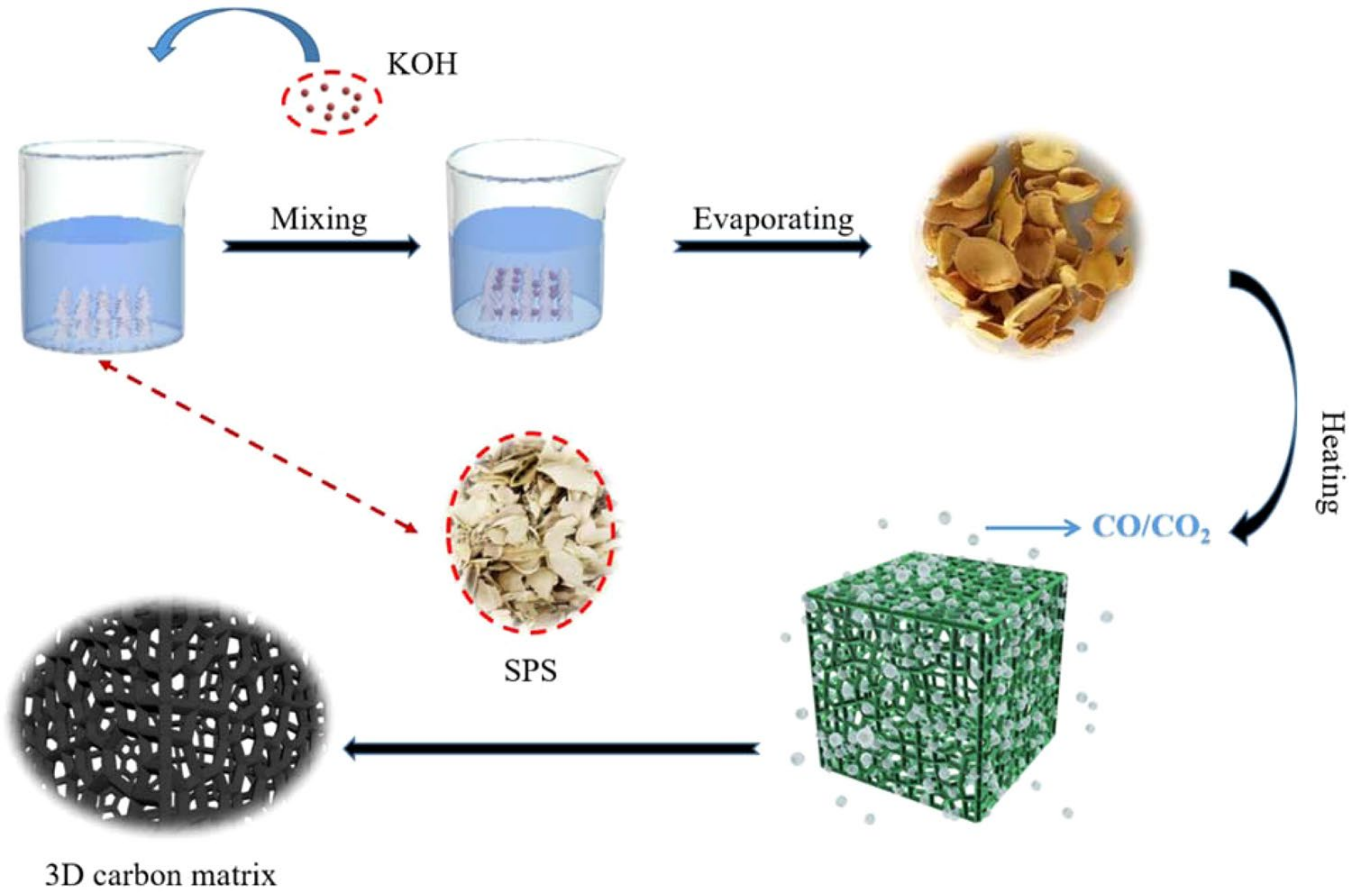
A schematic illustration of the synthesis for PC samples.

From Fig. 1, the SPS was firstly mixing with KOH. Then the ethanol slowly evaporates in a vacuum drying chamber, allowing the KOH to distribute evenly on the surface of the SPS. Subsequent heat treatments at 600 °C, 700 °C and 800 °C was carried out, allowing the following chemical reactions to occur.

1 $$6KOH+2C\to 2K+3{K}_{2}C{O}_{3}+3{H}_{2}$$

2 $${K}_{2}C{O}_{3}+2C\to 2K+3CO$$

3 $${K}_{2}O+C\to 2K+CO$$

4 $${K}_{2}C{O}_{3}\to {K}_{2}O+C{O}_{2}$$

5 $$C{O}_{2}+C\to 2CO$$

It is noteworthy that the CO and CO_2_ gas produced by the reaction (1)~(5) are came from the chemical etching process applied by KOH activator through heat treatments. And it is exactly that the etching process formed this hierarchical porous carbon.

**Figure 2:**
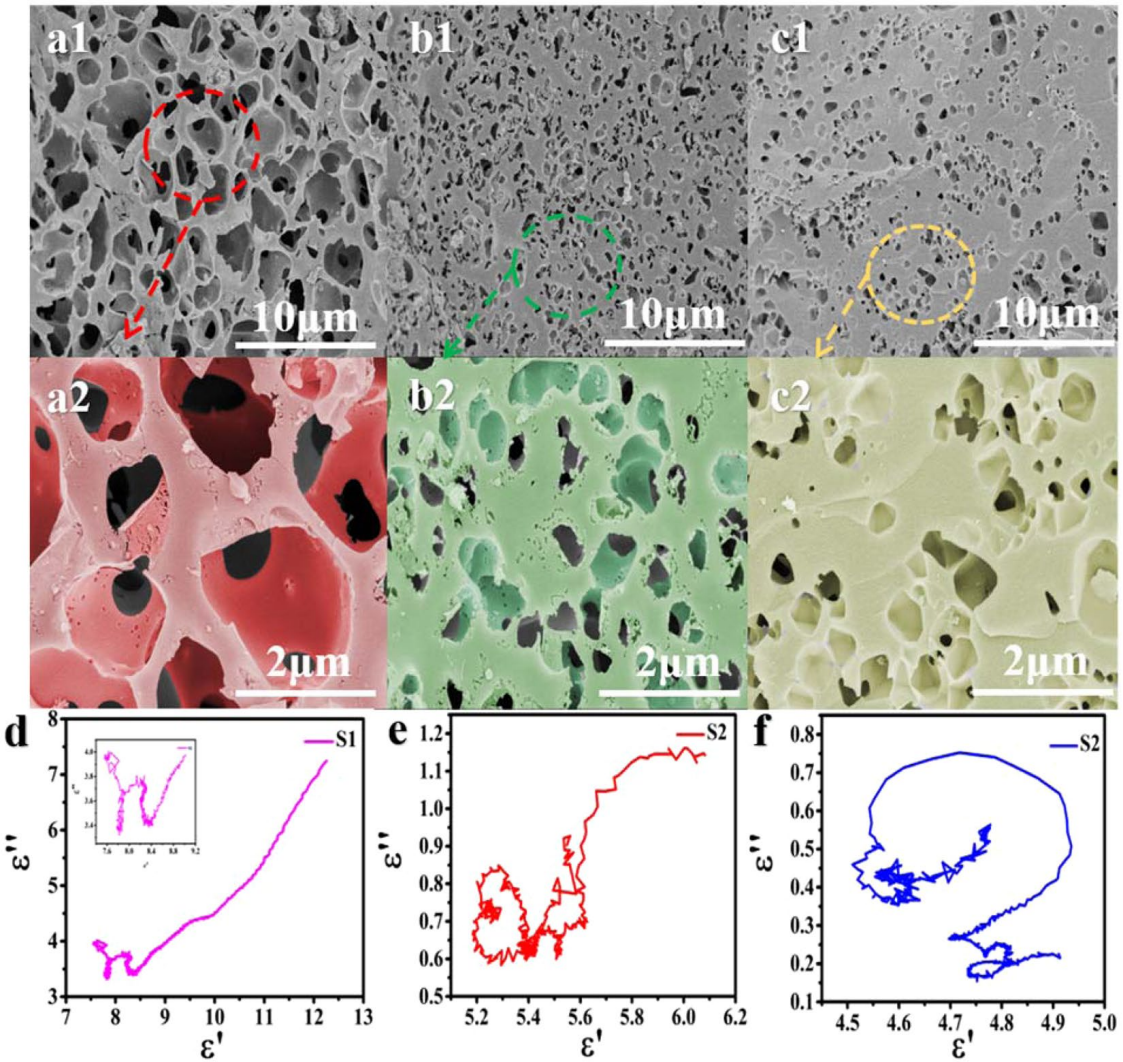
SEM images of (**a1**, **a2**) S1, (**b1**, **b2**) S2, and (**c1**, **c2**) S3 samples. Cole-Cole semicircle of (**d**) S1, (**e**) S2, (**f**) S3.

Through SEM images, we can clearly observe the micro-morphology of the sample (Fig. 2a–c). The obvious three-dimensional structure can be seen from Fig. 2a. By contrast, sample S2 (Fig. 2b) and S3 (Fig. 2c) possess relatively small pore size. Thus it can be concluded that the amount of activator added has a significant impact on the samples morphologies. Owing to the micropore existed in 3D porous structures, the space charge polarization occurred at the interfaces between the bio-carbon and the air make an important effect on the attenuation of electromagnetic wave. However, the resulting network does not allow microwaves to reflect multiple times in gigahertz, because the pore size is much smaller than the wavelength of the microwaves^24^. Nonetheless, the conductive network formed by this 3D structure greatly improves the conductive loss capacity of the sample^25^. The Cole-Cole hemicycles (Fig. 2d–f) could better prove the formed strong conductive loss capacity since the S1 sample tends to be a straight line at the end of the curve (Fig. 2c).

**Figure 3:**
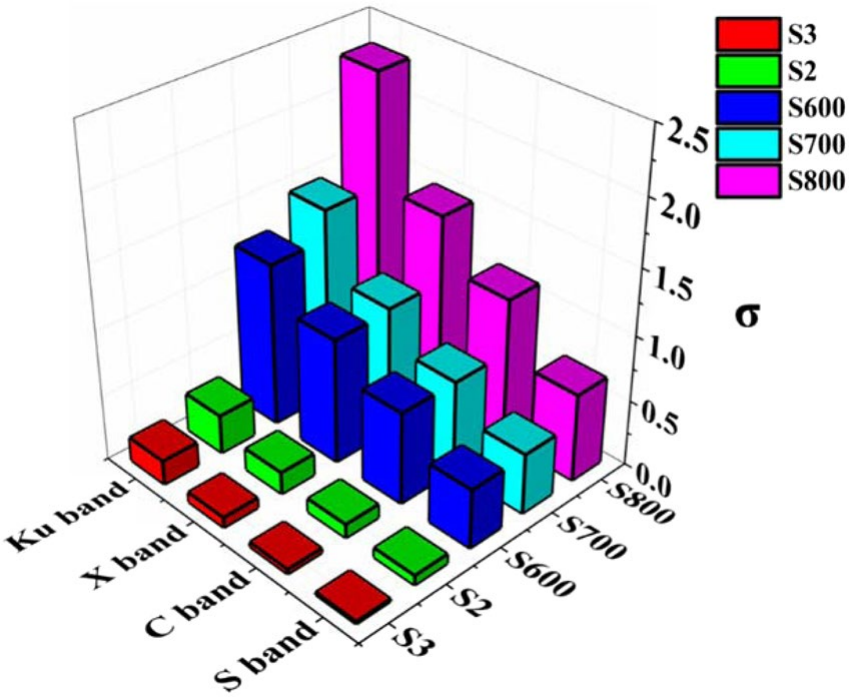
Comparison of electrical conductivity of all samples. (σ was calculated by the equation: σ = πε_0_ε″*f*, (ε_0_ = 8.55 × 10^−12^ F/m).

According to Eq. 6^26^:

6 $$\varepsilon ^{\prime\prime} =\frac{{\varepsilon }_{s}-{\varepsilon }_{\infty }}{1+{\omega }^{2}{\tau }^{2}}\omega \tau +\frac{\sigma }{\omega {\varepsilon }_{0}}$$

where σ, ω, and τ are electrical conductivity, angular frequency, and relaxation time, respectively. Hence, the ɛ″ is proportional to the sample’s conductivity. Figure 3 display the comparison of all samples’ electrical conductivity. When temperature rises the σ value increased significantly. At the same time the addition amount of KOH also has great impact on the electrical conductivity. Herein it can be deduced that temperature and the addition amount of activator could affect the samples electroconductivity.

Although the space charge polarization may exist, it is not the main reason for complex permittivity. From Fig. S1, one can find that the addition amount of the activator does not significantly affect the degree of graphitization. At the same time, the composition of the sample has no significant impact as well. All in all, the difference in the amount of KOH activator added caused the change of the morphology. The desirable 3D structures could be obtained by this simple method. Accordingly, the complex permittivity is controlled, which can be named as the Controlled-Morphology method.

Based on the transmission line theory, the EMW absorption properties could be assessed by the RL values, which are determined by the EM parameters according to following equations^27^:

7 $${Z}_{in}={Z}_{0}\sqrt{{\mu }_{r}/{\varepsilon }_{r}}\,\tanh [j(2\pi fd/c)]\sqrt{{\mu }_{r}{\varepsilon }_{r}}$$

8 $$RL(dB)=20\,\log |({Z}_{in}-{Z}_{0})/({Z}_{in}+{Z}_{0})|$$

where *Z*_0_ is the impedance of free air, *Z*_in_ is the input impedance of absorber, *d* is the thickness of absorber, and *c* is the velocity of light. In order to satisfy practical application, the RL values demand below −10 dB in a wide frequency range at thin thickness.

## Discussion

**Figure 4:**
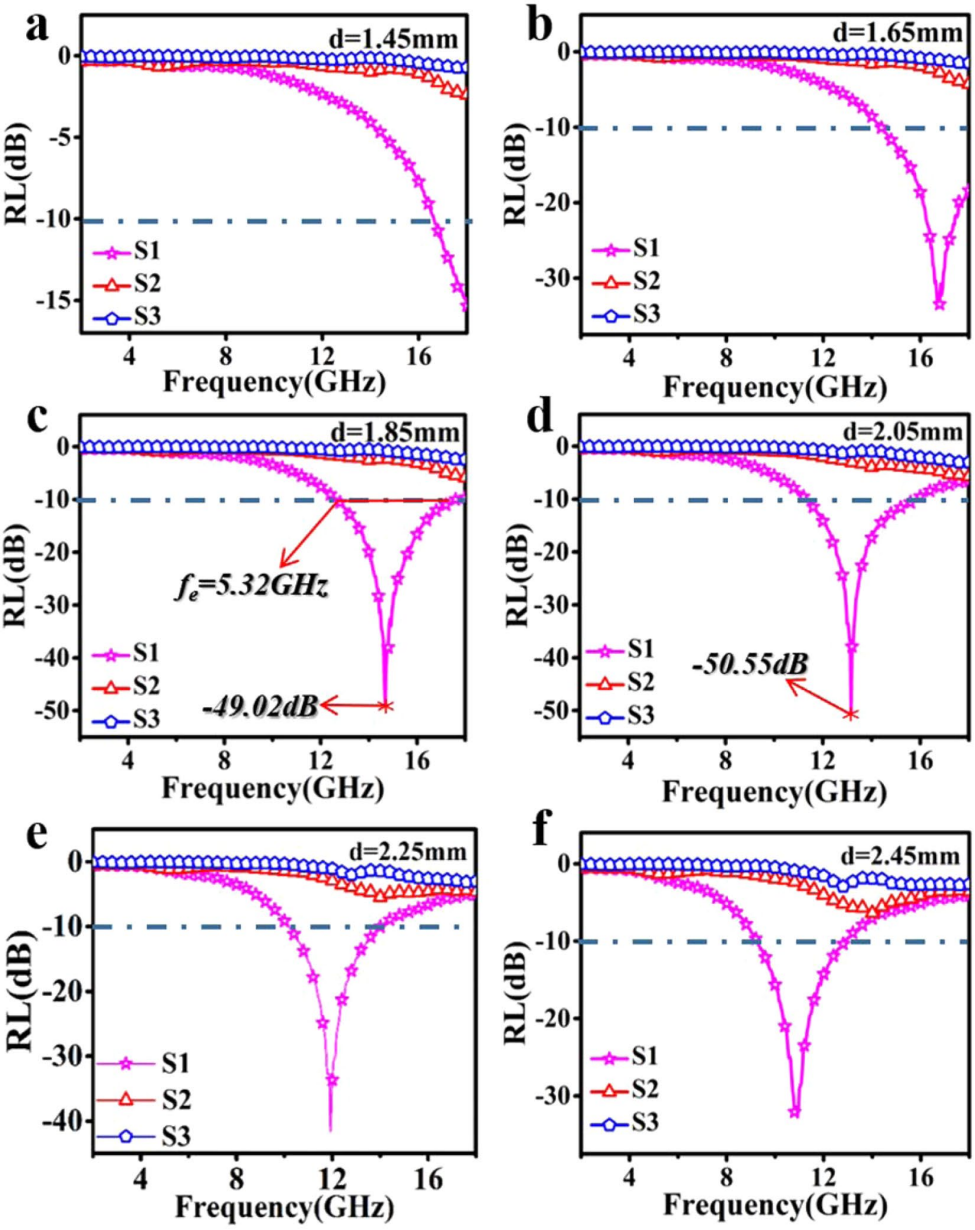
RL curves of three samples at different thickness of (**a**) 1.45 mm, (**b**) 1.65 mm, (**c**) 1.85 mm, (**d**) 2.05 mm, (**e**) 2.25 mm, (**f**) 2.45 mm.

Figure 4(a–f) represent RL curves at different thickness of S1, S2, S3, samples. On account of low complex permittivity, sample S2, S3 show no significant absorption properties (below −10 dB). In sharp contrast, sample S1 shows prominent microwave absorption (MA) performance at every thickness listed above. At the same time, as the thickness increases, the absorption gradually shifts to low frequency. The strong absorption intensity could reach −49.02 dB at 1.85 mm (Fig. 4c) and −50.55 dB at 2.05 mm (Fig. 4d), respectively. Beyond that, the maximum value of effective absorption bandwidth range (*f*_e_) reaches 5.32 GHz at thickness of 1.85 mm, covering the frequency range from 12.68 GHz to 18 GHz. In fact, the excellent MA performance of S2 compared with other samples is mainly attributed to its special micro-morphology. Moreover, It can be observed that the strongest absorption peaks gradually shift to lower frequencies as the thickness increases, which can be explained by quarter-wavelength cancellation model. When the incident and reflected electromagnetic waves differ π/4 at the phase angle, the reflected and incident waves completely cancel each other at the phase interface, and the RL reaches the minimum value. The relationship between RL peak value and matching frequency *f*_m_ and matching layer thickness *d*_m_ is shown in the following equation.

9 $${f}_{m}=\frac{c}{4{d}_{m}\sqrt{|{\mu }_{{\rm{r}}}|\times |{\varepsilon }_{r}|}}$$

Although the above work reflects the important position of conductive network in electromagnetic wave loss mechanism. In order to further optimize the absorbing performance, the influence of different factors on the loss ability of conductive network is explored. By controlling the annealing temperature, we conducted a series of characterization tests on the samples. Figure S2 shows a gradual ascend on complex permittivity when the heating temperature rises, which may result from the increased graphitization. Herein we named it as Graphitization-Controlled method. By using it, MA properties could be controlled.

**Figure 5:**
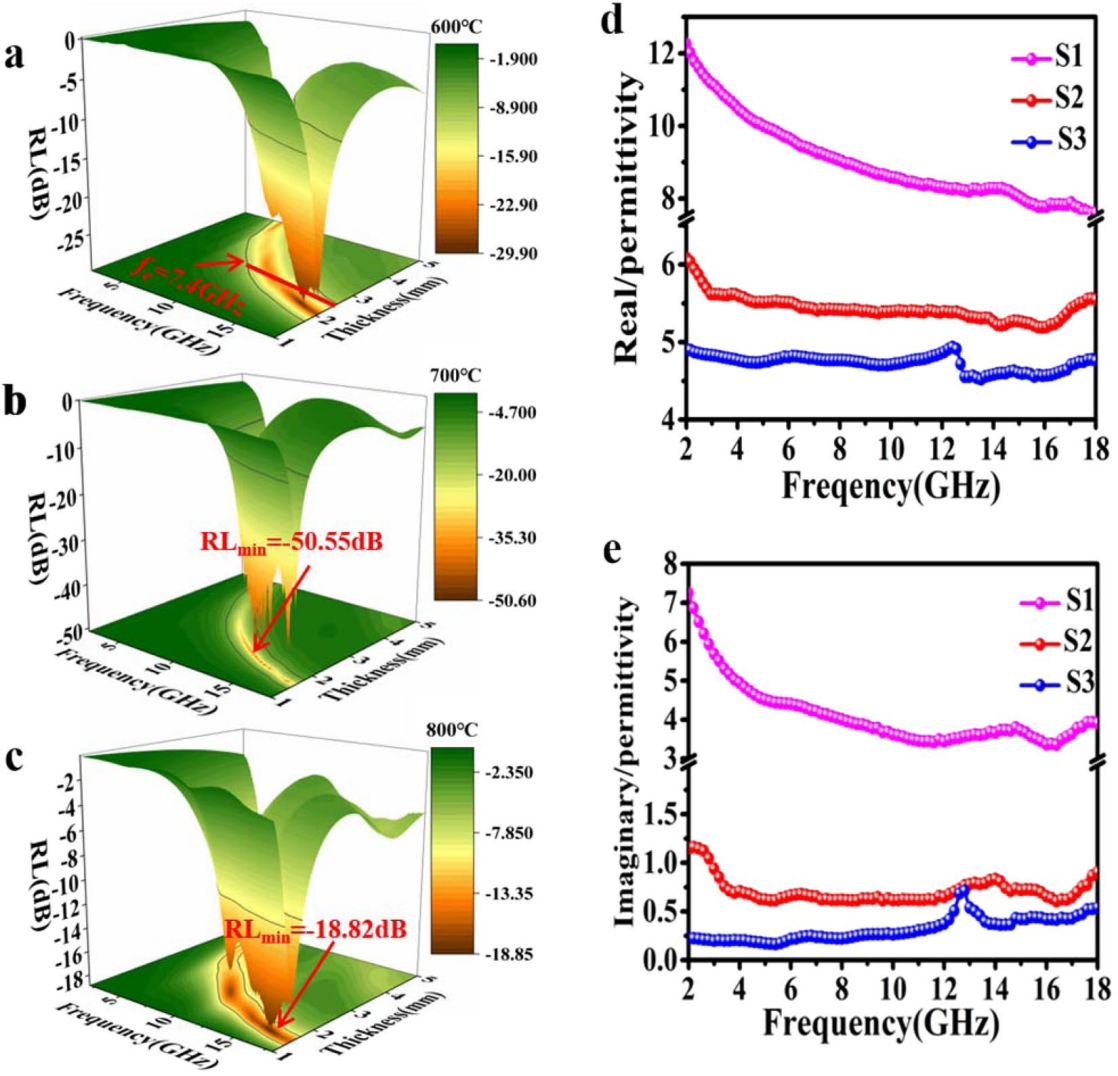
3D color map of MA performance in activation at 1:1 mass ratio under different calcination temperature of 600 °C (**a**), 700 °C (**b**), 800 °C (**c**). Real part (**d**) and imaginary part (**e**) of permittivity for S1, S2, S3 samples.

From Fig. 5a, sample S-600 shows a wide *f*_*e*_ bandwidth (from 10.6–18 GHz) when the sample thickness is about 2.5 mm, in which could cover the entire Ku band and the most X band. For sample S-700 (Fig. 5b), the strongest reflection loss is −50.55 dB at 13.16 GHz. While sample S-800 (Fig. 5c) shows both poor MA properties and narrow effective bandwidth, which may result from its exorbitant permittivity. Therefore, forming this unique 3D structure may be the primary cause for the relatively high complex permittivity of sample S1 (Fig. 5d,e).

A great deal of works has shown that the electromagnetic variation characteristics consist of two main parts^28^. They are respectively impedance matching and attenuation characteristics of the medium^29^. Impedance matching characteristics are defined by the following formula^30,31^:

10 $$Z=|{Z}_{in}/{Z}_{0}|$$

11 $${Z}_{in}={(\frac{{\mu }_{r}}{{\varepsilon }_{r}})}^{1/2}{Z}_{0}$$

where *Z*_in_ indicates the input impedance. *Z*_0_ stands for the free space impedance. *ε*_r_ on behalf of the complex permitivitty, and *μ*_r_ represents the complex permeability. In the ideal state, there isn’t any reflection between the air border and the absorber. By the formula (10–11), we know when *Z* value is near to 1, then *Z*_in_ needs to be approached to *Z*_0_. Also *ε*_r_ needs to be as close as possible to *μ*_r_. The excellent impedance matching makes sure the microwave can continue to get into the medium for dissipating.

**Figure 6:**
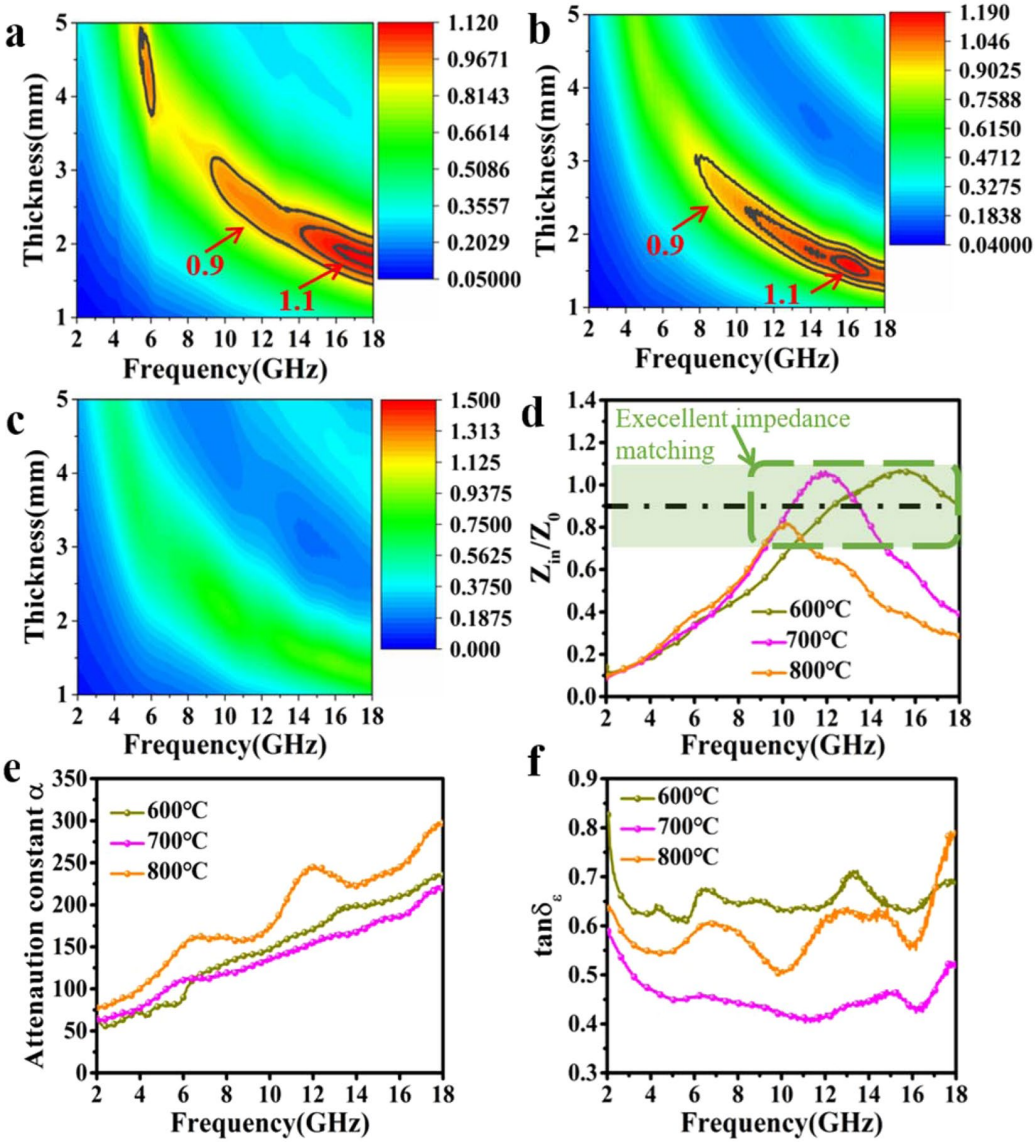
2D color fill images of *Z* values for S-600 (**a**), S-700 (**b**), S-800 (**c**). (**d**) Comparison of impedance matching at 2.1 mm of S-600, S-700, S-800. (**e**) Attenuation constant, and (**f**) the dielectric loss tangents.

In order to explore the mechanism of RL performance change, we calculated the impedance matching (*Z* value). For comparison, we used a 2D color fill images to present above results (Fig. 6a–c). It can be clearly observed, with the increase of heat treatment temperature, the impedance of the samples gradually turning into mismatch. The *Z*-value of S-600 sample (Fig. 6a) has a large area range in 0.9~1.1. However, the range of S-700 (Fig. 6b) is much narrower. When it comes to the S-800 sample (Fig. 6c), the *Z*-value in this range is no longer visible, which means most of the EMW was reflected and cannot be able to further dissipated in the absorber. Within the Z-value range of 0.7~1.1 (Fig. 6d), the entire frequency of the three samples has been covered from 9.24–18 GHz. The S-600 has the maximum coverage from 10.36 GHz to 18 GHz, which almost exactly corresponding to its *f*_e_. Indicating S-600 sample’s broad effective absorption bandwidth derives from its excellent impedance matching characteristics in the wide frequency band.

Normally we evaluate the dielectric loss capacity of a certain material by calculating the attenuation constant (*α*) and the dielectric relaxation tangent (tan *δ*_ε_). They are expressed as following formulation^32,33^:

12 $$\tan \,{\delta }_{\varepsilon }=\varepsilon ^{\prime\prime} /\varepsilon ^{\prime} $$

13 $$\alpha =\frac{\sqrt{2}\pi f}{c}\times \sqrt{(\mu ^{\prime\prime} \varepsilon ^{\prime\prime} -\mu ^{\prime} \varepsilon ^{\prime} )+\sqrt{{(\mu ^{\prime\prime} \varepsilon ^{\prime\prime} -\mu ^{\prime} \varepsilon ^{\prime} )}^{2}+{(\mu ^{\prime} \varepsilon ^{\prime\prime} -\mu ^{\prime\prime} \varepsilon ^{\prime} )}^{2}}}$$

Figure 6e,f shows the tan *δ*_ε_ and *α* values of three samples between 2 and 18 GHz. Clearly, S-800 sample has a higher dielectric loss capacity, which possess the limited impedance matching. On the contrary, the S-600 and S-700 samples not only possess perfect dielectric loss capacity, but also have excellent impedance matching characteristics. Both those two characteristics contribute to great potential in EMW absorption of the S-600 and S-700 samples.

**Figure 7:**
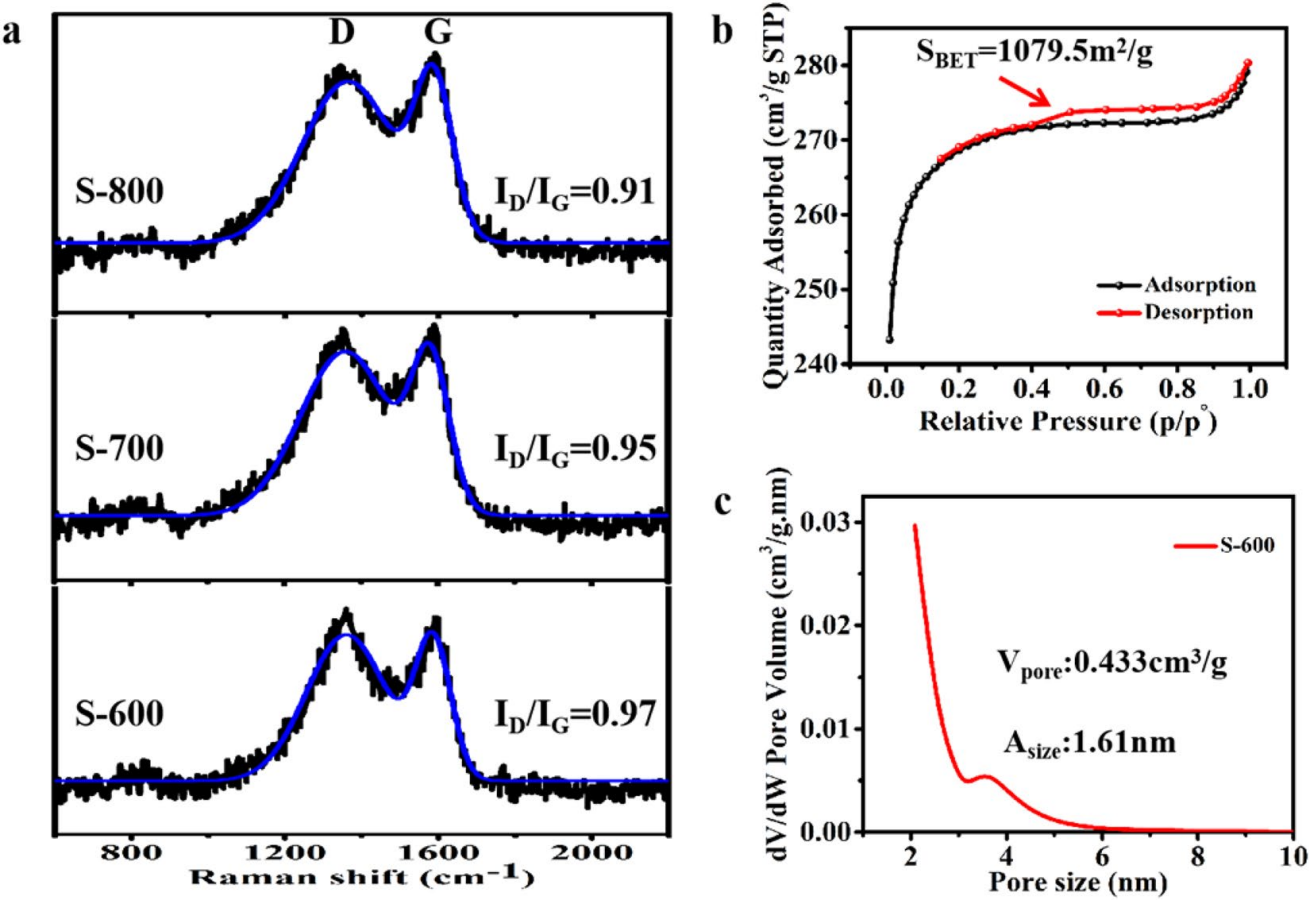
Raman spectra of S-600, S-700, S-800 (**a**), nitrogen sorption isotherm (**b**) and size distribution (**c**) of S-600 sample.

In order to prove the conjecture mentioned above (heating temperature induced increased graphitization), samples of S-600, S-700 and S-800 were analyzed by Raman spectroscopy (Fig. 7a). It can be found that the R value (I_D_/I_G_) of S-600 was the highest at 0.97, while that of S-800 was the lowest at 0.91. A clear downward trend appeared. Generally, this test method is used to characterize the graphitization degree of samples. Peak D and peak G are Raman characteristic peaks of C atomic crystal, which are around 1300 cm^−1^ and 1580 cm^−1^, respectively. It is well known that peak D and peak G represent the lattice defect of atom C and the in-plane stretching vibration of sp^2^ hybridization of carbon atom, respectively. Therefore, higher R value indicates more lattice defects of C atom, and higher graphitization degree on the contrary. Herein the S-600 has the lowest graphitization, and the S-800 has the highest graphitization.

Figure 7b gives the nitrogen adsorption isotherm curve of S-600 sample, from left to right, it has an obvious adsorption process at a relatively low pressure, while a significant hysteresis loop appears at a relatively high pressure. This indicates that both micropores and mesoporous pores exist. Besides, the sample has a specific surface area (S_BET_) of 1079.5 m^2^/g, verifying tremendous amounts of pores distributed, forming its intrinsic porous structure. Figure 7c shows the aperture distribution curve of S-600. A cliff fall means that most of the nanopores in this sample are distributed around 2 nm in diameter. The average pore size and pore volume were 1.66 nm, 0.433 cm^3^/g, respectively.

**Figure 8:**
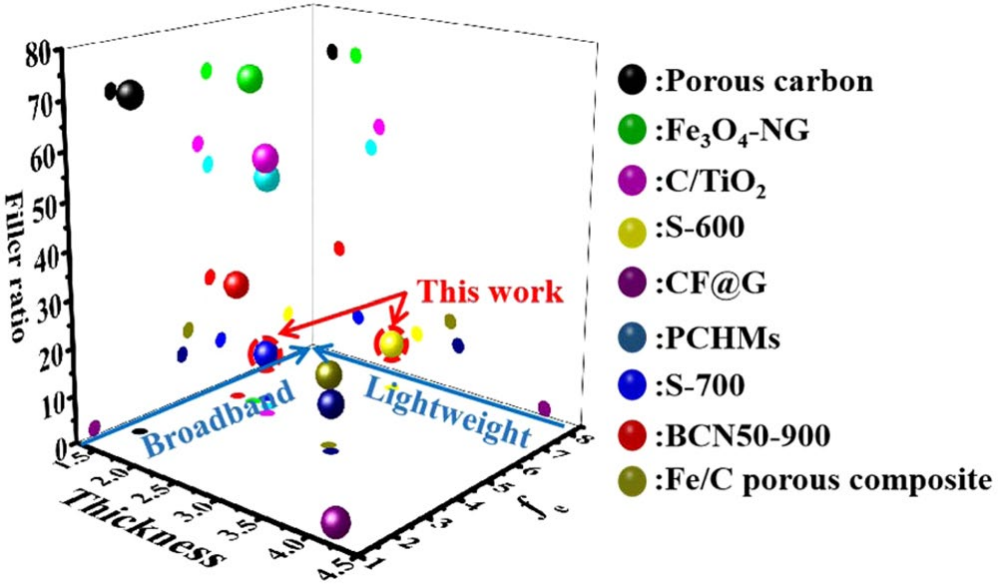
EMW absorption properties of recently reported carbon-based absorbing materials and this work.

From Fig. 8, it can be seen that our samples show great advantages in filler ratio (≤10%), thickness (<2 mm) and bandwidth (7.4 GHz). This excellent performance is mainly deriving from the large number of holes of various sizes range distributed in the samples. The presence of micropores and mesopores not only greatly improves the contact surface with air, increasing the space charge polarization, but also reduces the bulk density of those samples. In addition, the macropore with large aperture provide a good conductive network, so that free charge can form a loop current on it, to improve conductive loss ability.

## Conclusions

In this work, a kind of porous carbon by activating biomass with high specific surface area, low density and strong dielectric property was obtained. The dielectric properties and morphologies were optimized by controlling the dosage of activator and the heating temperature. It can be concluded that the exotic 3D mesh structure and excellent circuit network are good for strong conductivity loss. Meanwhile, the existence of porous structure also promotes the space charge polarization, and these loss factors integrate with each other to promote the EMW absorption. With a low fill ratio of only 10%, the RL_min_ reaches −50.55 dB and the widest *f*_e_ is 7.4 GHz. When the thickness is 1.85 mm, the maximum bandwidth can also achieve 5.32 GHz. In conclusion, this biomass derived carbon not only has the characteristics of low price cost and simple process, but also shows the excellent performance of absorbing materials.

## Method

### Materials

The shell of pumpkin seeds (SPS) were recycled from kitchen waste. In order to wipe off the impurities, the SPS have been washed three times with ethanol and distilled water, respectively. Potassium hydroxide (KOH) and hydrochloric acid (HCl) were bought from the Nanjing chemical reagent Co., LTD. All the chemical materials were analytically pure and no procedure has been made for further purification.

### Preparation of PC specimens

The as-cleaned SPS and KOH were added into 20 mL ethanol at 1:1, 2:1, 3:1 mass ratio. Then they were dried in a vacuum oven at 60 °C until the ethanol completely evaporated, so that KOH was evenly distributed in SPS. Afterwards, the processed samples were sent to the tubular furnace for pyrolysis at 700 °C, with nitrogen as the protective atmosphere. The heating rate is 2 °C/s and the temperature is kept for 2 h. Finally, the resulting products were ground into powder with an agate mortar, dissolved in distilled water and pH adjusted by HCl to neutral, filtered and dried. The as-prepared samples at 1:1, 1:2, 1:3 mass ratio were named S1 (S-700), S2, and S3, respectively. The pyrolysis temperature at 600 °C and 800 °C is the same as the above procedure. They were named as S-600 (600 °C) and S-800 (800 °C), when the mass ratio was 1:1.

### Characterization

XRD testing was implemented using a Bruker D8 ADVANCE diffractometer to identify the phase structure of specimens. The sample’s microstructure was observed by a Hitachi S4800 field-emission scanning electron microscopy (SEM). Raman spectrum was collected via a Renishaw inVia 2000 Raman microscope. The special surface area and pore size was identified by the Nitrogen isothermal adsorption-desorption analyzer (Micromeritics ASAP 2010). The EM parameters were tested by vector network analyzer (Agilent PNA N5244A). The toroidal ring samples were prepared by mixing paraffin with as-prepared powders (10 wt %) and then pressed into a mold with φ_out_ of 7.00 mm and φ_in_ of 3.04 mm.

## Supplementary information

A biomass derived porous carbon for broadband and lightweight microwave absorption

## References

[1] Quan B (2019). Defect Engineering in Two Common Types of Dielectric Materials for Electromagnetic Absorption Applications. Adv. Funct. Mater..

[2] Cheng Y (2018). The outside-in approach to construct Fe_3_O_4_ nanocrystals/mesoporous carbon hollow spheres core−shell hybrids toward microwave absorption. ACS Sustainable Chem. Eng..

[3] Lv HL (2019). A flexible microwave shield with tunable frequency-transmission and electromagnetic compatibility. Adv. Funct. Mater..

[4] Lee SH, Kang D, Oh IK (2017). Multilayered graphene-carbon nanotube-iron oxide three-dimensional heterostructure for flexible electromagnetic interference shielding film. Carbon.

[5] Lv HL, Ji GB, Liang XH, Zhang HQ, Du YW (2015). A novel rod-like MnO_2_@Fe loading on graphene giving excellent electromagnetic absorption properties. J. Mater. Chem. C.

[6] Cheng Y (2018). Rationally regulating complex dielectric parameters of mesoporous carbon hollow spheres to carry out efficient microwave absorption. Carbon.

[7] Pu J, Kong W, Lu CC, Wang ZH (2015). Directly carbonized lotus seedpod shells as high-stable electrode material for supercapacitors. Ionics.

[8] Hou JH, Cao CB, Idrees F, Ma XL (2015). Hierarchical porous nitrogen-doped carbon nanosheets derived from silk for ultrahigh-capacity battery anodes and supercapacitors. ACS Nano.

[9] Cao H (2016). A shiitake-derived nitrogen/oxygen/phosphorus co-doped carbon framework with hierarchical tri-modal porosity for high-performance electrochemical capacitors. RSC Adv..

[10] Liu Y (2016). Preparation of activated carbon from willow leaves and evaluation in electric double-layer capacitors. Mater. Lett..

[11] Zhao HQ (2019). Biomass-Derived Porous Carbon-Based Nanostructures for Microwave Absorption. Nano-Micro Lett..

[12] Zhang L (2013). High-performance supercapacitor electrode materials prepared from various pollens. Small.

[13] Xu Q (2017). Watermelon-inspired Si/C microspheres with hierarchical buffer structures for densely compacted lithium-ion battery anodes. Adv. Energy. Mater..

[14] Qian WJ (2014). Human hair-derived carbon flakes for electrochemical supercapacitors. Energy Environ. Sci..

[15] Gao SY, Li XG, Li LY, Wei XJ (2017). A versatile biomass derived carbon material for oxygen reduction reaction, supercapacitors and oil/water separation. Nano Energy.

[16] Zheng YH, Wang YS, Lu YX, Hu YS, Li J (2017). A high-performance sodium-ion battery enhanced by macadamia shell derived hard carbon anode. Nano Energy.

[17] Wang Z (2018). Pomelo peels-derived porous activated carbon microsheets dual-doped with nitrogen and phosphorus for high performance electrochemical capacitors. J. Power Sources.

[18] Shu CY (2018). Mesoporous 3D nitrogen-doped yolk-shelled carbon spheres for direct methanol fuel cells with polymer fiber membranes. Carbon.

[19] Chang BB (2015). SO_3_H-functionalized hollow mesoporous carbon sphere prepared by simultaneously achieving sulfonation and hollow structure. J. Porous Mater..

[20] Zhang N (2017). Nitrogen–phosphorus co-doped hollow carbon microspheres with hierarchical micro–meso–macroporous shells as efficient electrodes for supercapacitors. J. Mater. Chem. A.

[21] Xu F, Lin TQ, Bi H, Huang FQ (2017). Graphene-like carbon with three-dimensional periodicity prepared from organic-inorganic templates for energy storage application. Carbon.

[22] Yao YP (2019). The Relations between Minor Components and Antioxidant Capacity of Five Fruits and Vegetables Seed Oils in China. J. Oleo Sci..

[23] Zhao HQ, Cheng Y, Lv HL, Ji GB, Du YW (2019). A novel hierarchically porous magnetic carbon derived from biomass for strong lightweight microwave absorption. Carbon.

[24] Gu WH (2019). Composition and Structure Design of Co_3_O_4_ Nanowires Network by Nickel Foam with Effective Electromagnetic Performance in C and X Band. ACS Sustainable Chem. Eng..

[25] Zhao HQ (2018). Achieving Sustainable Ultralight Electromagnetic Absorber from Flour by Turning Surface Morphology of Nanoporous Carbon. ACS Sustainable Chem. Eng..

[26] Cheng Y (2019). Engineering morphology configurations of hierarchical flower-like MoSe_2_ spheres enable excellent low-frequency and selective microwave response properties. Chem. Eng. J..

[27] Yang ZH, Lv HL, Wu RB (2016). Rational construction of graphene oxide with MOF derived porous NiFe@C nanocubes for high-performance microwave attenuation. Nano Res..

[28] Cao MS (2018). Thermally Driven Transport and Relaxation Switching Self-Powered Electromagnetic Energy Conversion. Small.

[29] Ye F (2018). Direct Growth of Edge-Rich Graphene with Tunable Dielectric Properties in Porous Si_3_N_4_ Ceramic for Broadband High-Performance Microwave Absorption. Adv. Funct. Mater..

[30] Liang XH (2017). Tunable Dielectric Performance Derived from the Metal−Organic Framework/Reduced Graphene Oxide Hybrid with Broadband Absorption. ACS Sustain. Chem. Eng..

[31] Lv HL, Ji GB, Liu W, Zhang HQ, Du YW (2015). Achieving hierarchical hollow carbon@Fe@Fe_3_O_4_ nanospheres with superior microwave absorption properties and lightweight features. J. Mater. Chem. C.

[32] Lv HL, Zhang HQ, Zhao J, Ji GB, Du YW (2016). Achieving excellent bandwidth absorption by a mirror growth process of magnetic porous polyhedron structures. Nano Res..

[33] Lou ZC (2019). Synthesis of porous carbon matrix with inlaid Fe_3_C/Fe_3_O_4_ micro-particles as an effective electromagnetic wave absorber from natural wood shavings. J. Alloys Compd..

