# Supplementary material for: A biomass derived porous carbon for broadband and lightweight microwave absorption
Source: Sci Rep. 2019 Dec 9;9:18617. doi: 10.1038/s41598-019-54104-2 (PMC6901585; doi:10.1038/s41598-019-54104-2)
Supplement: Supplementary file 1 — A biomass derived porous carbon for broadband and lightweight microwave absorption [file 41598_2019_54104_MOESM1_ESM.doc]

A biomass derived porous carbon for broadband and lightweight microwave absorption

Zhu Zhang, ^1, 2^ Huanqin Zhao, ^2^ Weihua Gu, ^2^ Lieji Yang, ^2^ Baoshan Zhang，^1, *^

^1^ School of Electronic Science and Engineering, Nanjing University, Nanjing 210093, P. R. China

^2^ College of Materials Science and Technology, Nanjing University of Aeronautics and Astronautics, Nanjing 211100, P. R. China.

*Corresponding Author

Prof. Dr. Baoshan Zhang

Tel: +86-25-83597192

E-mail: bszhang@nju.edu.cn

Supporting Information


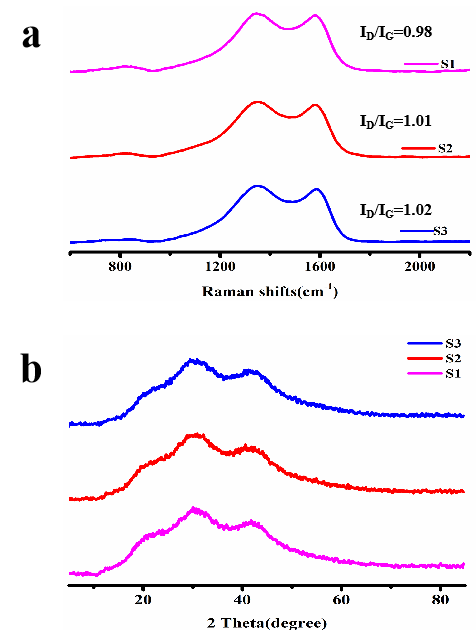


**Figure S1**. (a) Raman spectra and (b) XRD profiles of S1, S2, S3, samples


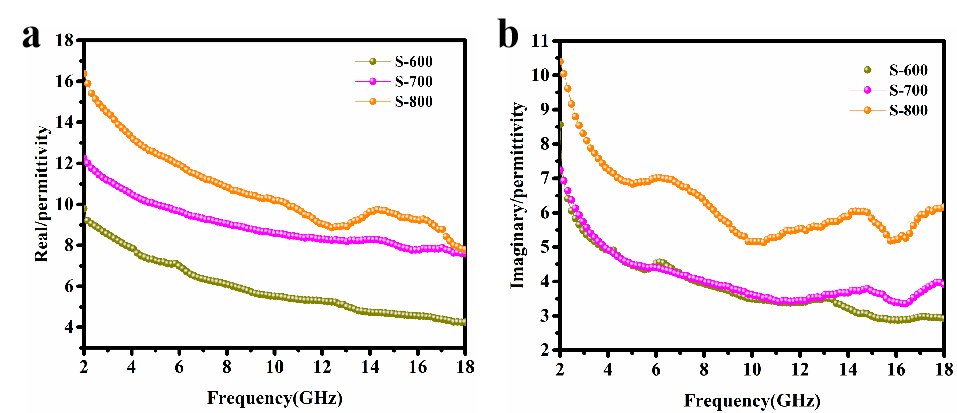


**Figure S2.** (a) Real part (a) and imaginary part (b) of permittivity of S-600, S-700, S-800, samples.
